# Supplementary material for: Relating sex-bias in human cortical and hippocampal microstructure to sex hormones
Source: Nat Commun. 2024 Aug 23;15:7279. doi: 10.1038/s41467-024-51459-7 (PMC11344136; doi:10.1038/s41467-024-51459-7)
Supplement: Supplementary file 1 — Supplementary Information [file 41467_2024_51459_MOESM1_ESM.pdf]

Supplement: Relating sex-bias in human cortical and hippocampal microstructure to sex hormones

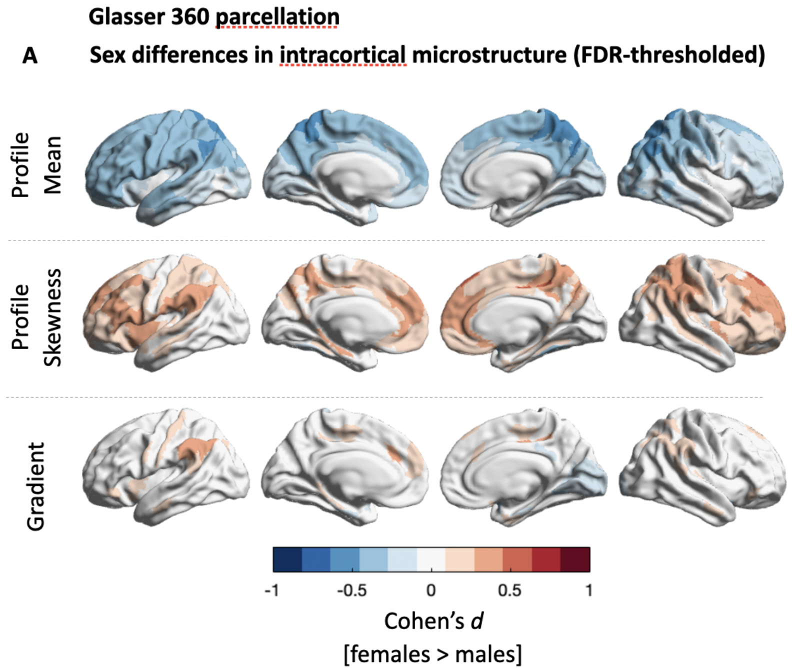

Supplement 1. Intracortical T1w/T2w signal intensity profiling sex difference, Glasser 360 parcellation. Shown are Cohen's d values for the (females (n = 594) > males (n = 499)) contrast, controlling for family structure (including the interaction between twin status and family status), only coloring in parcels with a p-value lower than the FDR threshold. Source data are provided as a Source Data file.

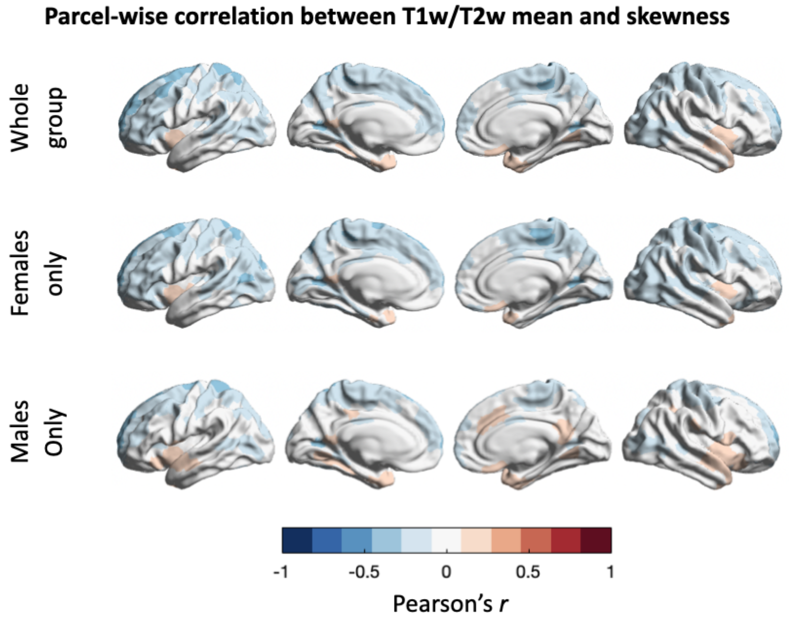

Supplement 2. Parcel-wise correlation between baseline T1w/T2w mean and skewness profiles, for the whole group (n = 1093), for females (n=594) only and for males only (n = 499). Red areas represent

positive correlation between skewness and mean T1w/T2w, blue represent negative correlations. Source data are provided as a Source Data file.

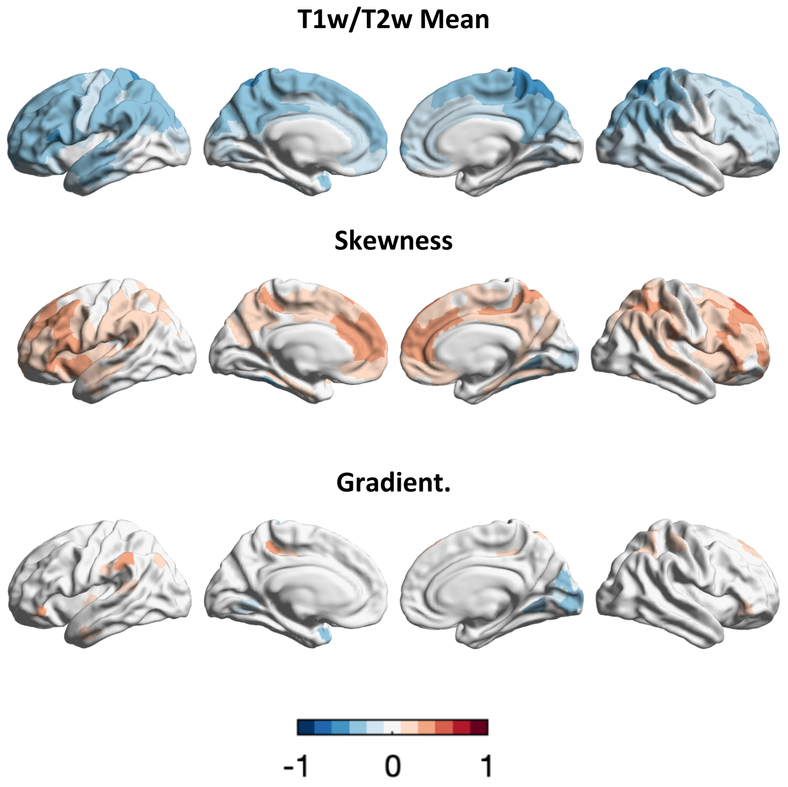

Supplement 3. Intracortical T1w/T2w signal intensity profiling with additional covariates. Shown are z-values for the (females (n = 594) > males (n = 499)) contrast, controlling for family structure (including the interaction between twin status and family status) and cortical thickness, FDR controlled Cohen's d. Blue shades represent Cohen's d < 0, indicating higher values in males; red shades represent Cohen's d > 0, indicating higher values in females. Source data are provided as a Source Data file.

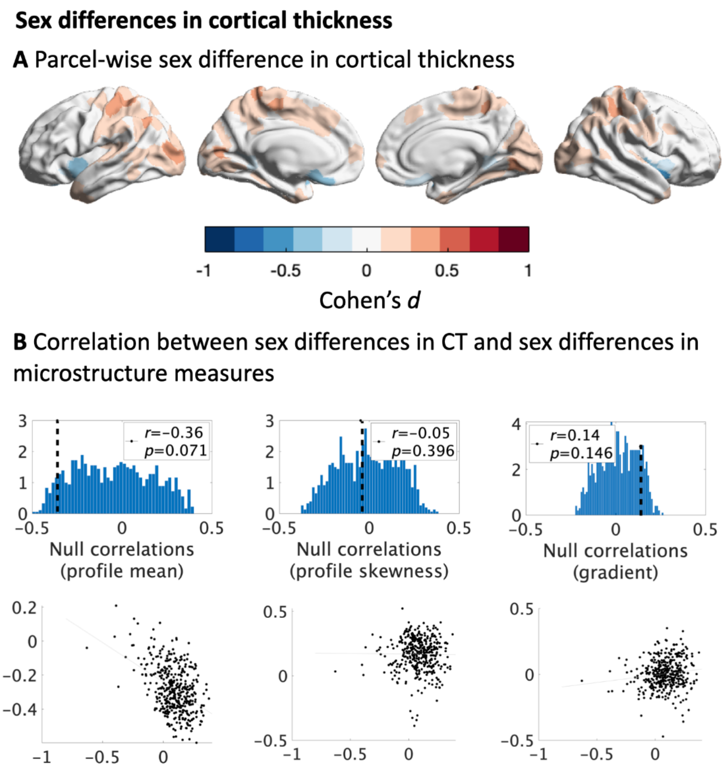

Supplement 4. Associations between cortical thickness and microstructural sex differences. (A) FDR-thresholded Cohen's d maps showing significant sex differences (females (n = 594) > males (n = 499)) in cortical thickness, Red colors represent microstructural values were higher for females, blue represent values higher for males. B) Associations between sex differences in cortical thickness and effect values (Cohen's d for each of the 400 Schaefer parcels) for each of the T1w/T2w profile-based intracortical measures. The upper row visualizes zero-distributions between random hierarchies and effect maps in comparison to the statistical r-value, the bottom row plots cortical thickness sex differences on the X-axis, and sex differences of microstructural measures on the Y-axis. Source data are provided as a Source Data file.

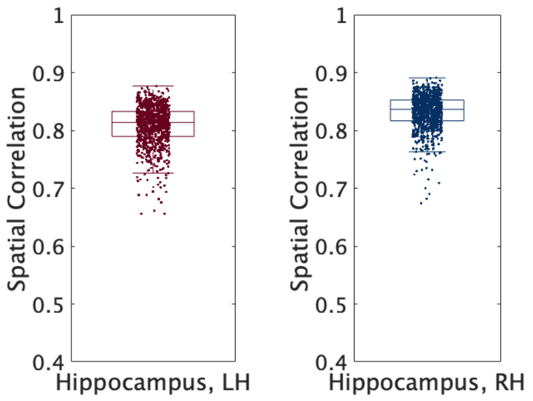

Supplement 5. Internal Consistency Hippocampus. Boxplots represent Pearson's r-values between unthresholded t-statistics resulting from two respective split-halves of the sample (n = 1000 permutations) comparing the microstructural mean of the left and right hippocampus between females and males, indicating their reliability. The median is shown as the central mark, the box indicates 25th and 75th percentile; whiskers include all values not considered outliers (1.5*IQR from the quartile). Source data are provided as a Source Data file.

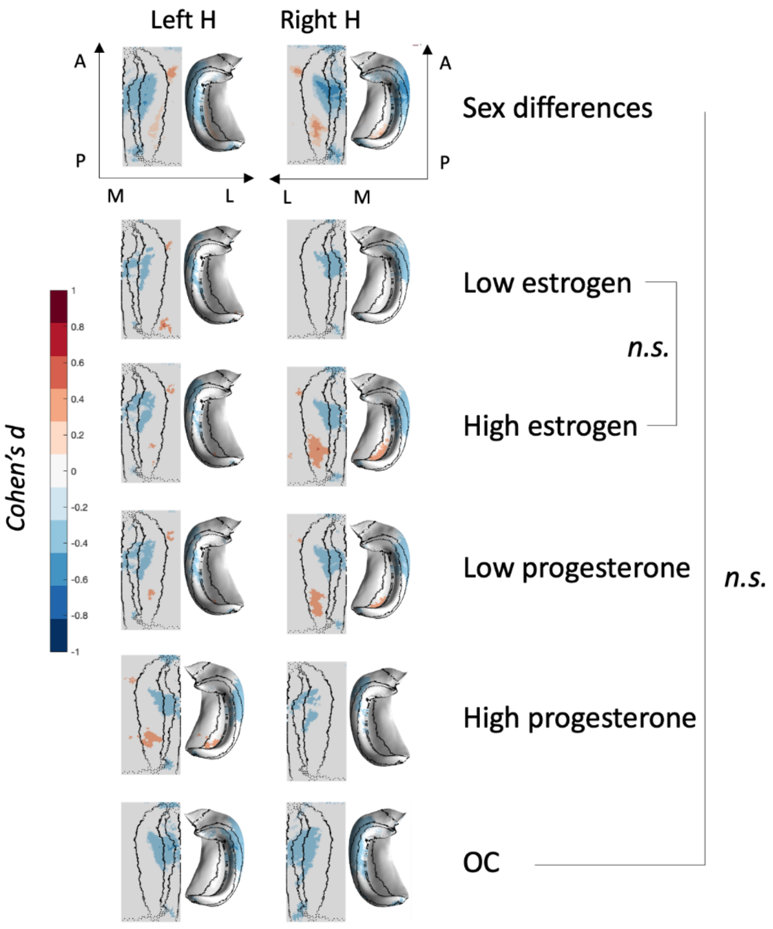

Supplement 6. FDR-thresholded Cohen's d maps of T1w/T2w profile mean between males and subsamples of females (females > males) divided by OC use and menstrual cycle phase projected on the unfolded hippocampus. On average, all effects are different from each other. Brackets (n.s.) on the right show where this is not the case, i.e. where the average effect across vertices replicates. Source data are provided as a Source Data file.

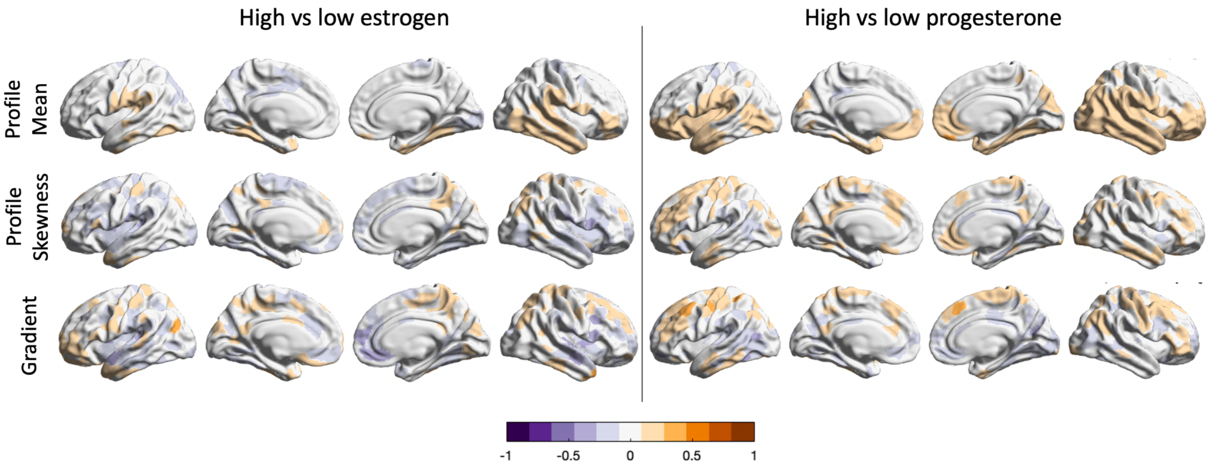

Supplement 7. Non-significant microstructural differences between NC females (Cohen's d). NC females (n = 284) were divided by hormone estimations according to self-reported days after menstruation. Columns are the three microstructural measures T1w/T2w mean, T1w/T2w skewness, and the microstructural gradient. Purple areas are parcels which had higher values for females in the high estrogen (n = 184) or high progesterone (n = 113) group, orange showshad higher values for NC females in the respective lower hormonal group (all Cohen's d; $n_{\text{low estrogen}}$ = 100; $n_{\text{low progesterone}}$ = 171). Note that no parcel was significant at an FDR-threshold. Source data are provided as a Source Data file.

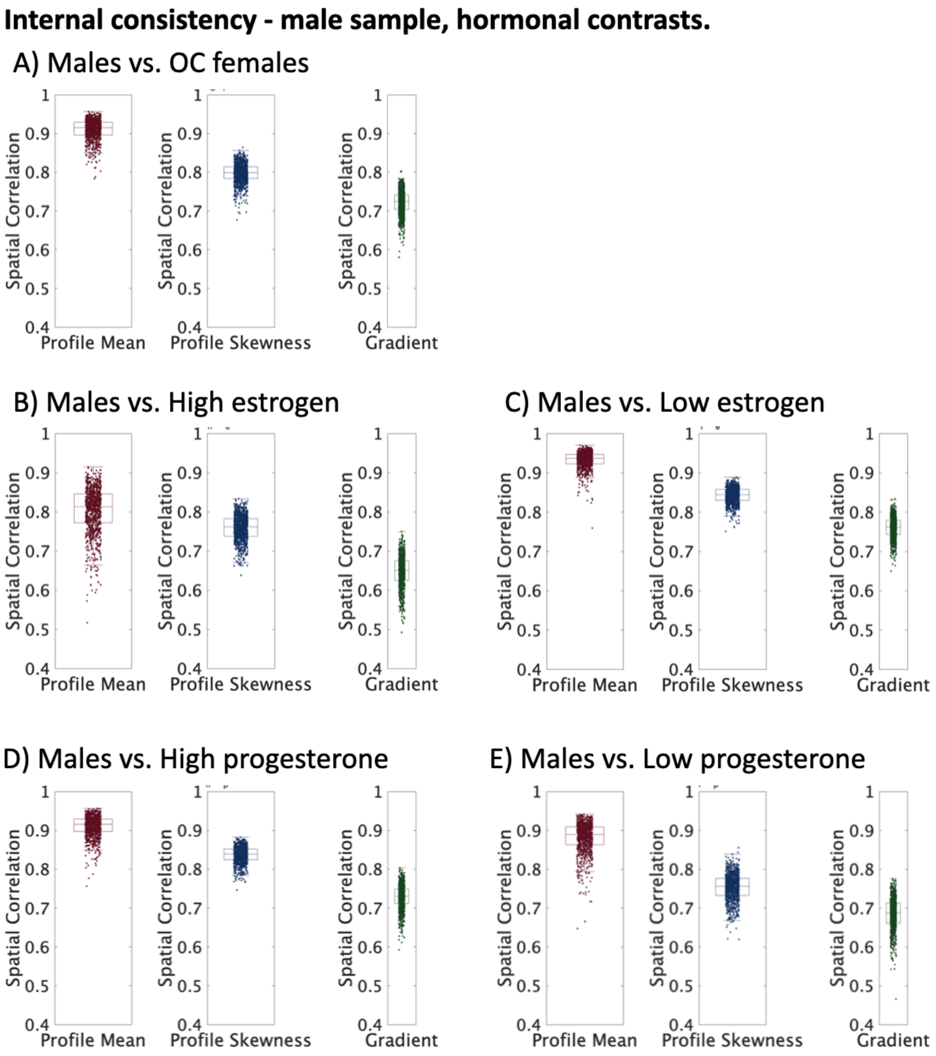

Supplement 8. Split-correlation of 1000 random permutations for all hormonal contrasts and each microstructural measure. For every split, we computed the contrast between males and females, randomly choosing only a subsample of males, such that $n_{(\text{males})} = n_{(\text{females})}$. We did so matching $n_{(\text{males})}$ to $n_{(\text{OC females})}$ = 170 in A), $n_{(\text{males})}$ to $n_{(\text{High estrogen females})}$ = 184 in B), $n_{(\text{males})}$ to $n_{(\text{Low estrogen females})}$ = 100 in C), $n_{(\text{males})}$ to $n_{(\text{High progesterone females})}$ = 113 in D) and $n_{(\text{males})}$ to $n_{(\text{Low progesterone females})}$ = 171 in E). We then computed the internal consistency for this randomly chosen male subsample by correlating the effect sizes of this contrast with the Cohen's d effect sizes of an equally sized and randomly chosen subsample of males. Datapoints represent correlation values (Pearson's r) for each split. The median

is shown as the central mark in each subpanel, the box indicates 25th and 75th percentile; whiskers include all values not considered outliers (1.5*IQR from the quartile). Source data are provided as a Source Data file.

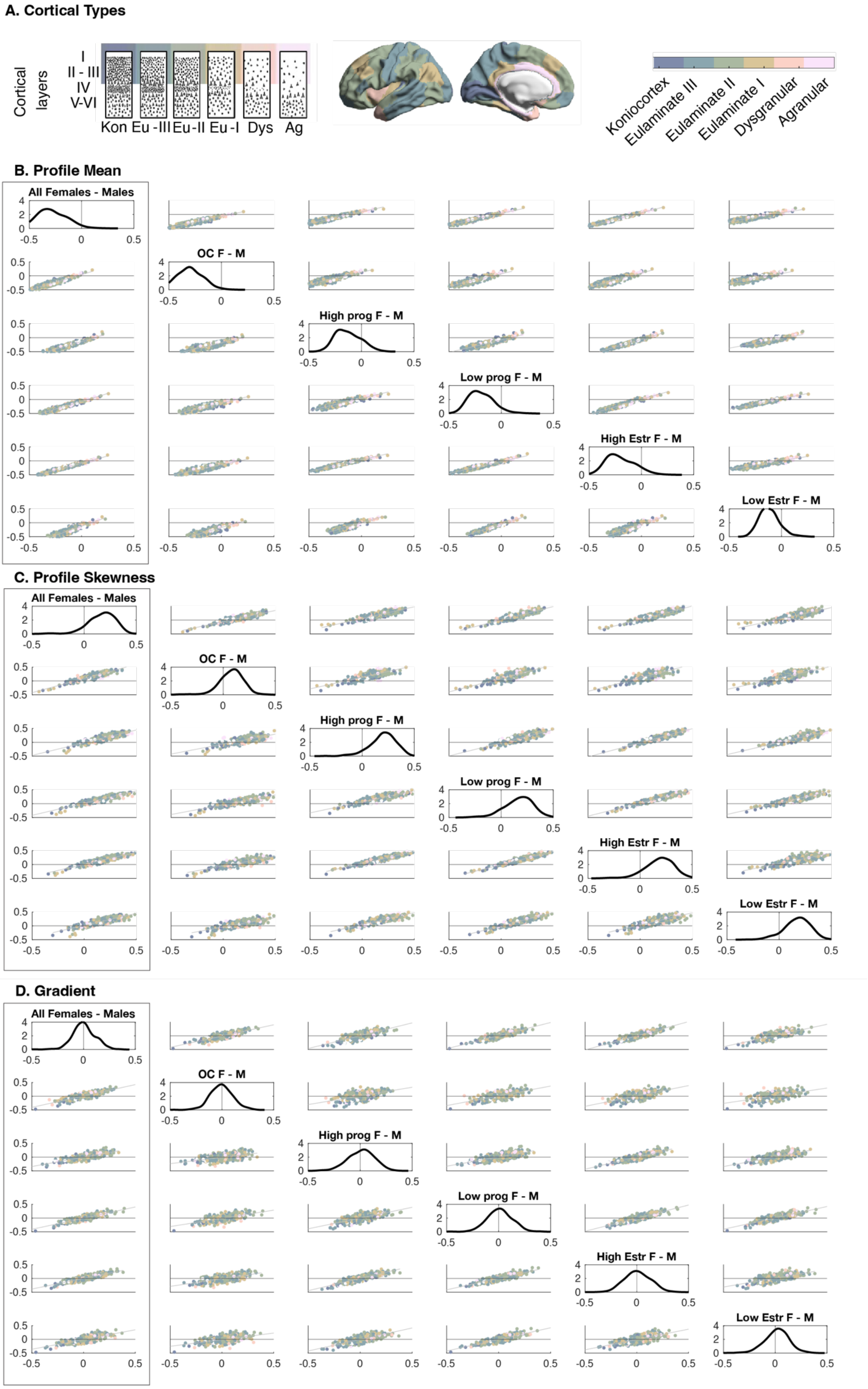
A. Cortical Types
Cortical layers
I
II - III
IV
V-VI
Kon Eu -III Eu-II Eu -I Dys Ag
Koniocortex
Eulaminate III
Eulaminate II
Eulaminate I
Dysgranular
Agranular
B. Profile Mean
All Females - Males
OC F - M
High prog F - M
Low prog F - M
High Estr F - M
Low Estr F - M
C. Profile Skewness
All Females - Males
OC F - M
High prog F - M
Low prog F - M
High Estr F - M
Low Estr F - M
D. Gradient
All Females - Males
OC F - M
High prog F - M
Low prog F - M
High Estr F - M
Low Estr F - M

Supplement 9. Effect sizes for sex differences in T1w/T2w profile mean (B), skewness (C) and microstructural gradient (D) for each of n=400 Schaefer parcel, and how these effects change depending on which female subgroups males are compared to, each parcel colored by cortical type (A). The diagonal shows the sex-different effect size distributions per comparison, and how they shift depending on the contrast. Scatter plots show correlation between two respective effects, and the deviance of each parcel from the other contrast's effect size. The first column (black box) is the original all females (n = 594) vs. all males (n = 499) sex difference effect, compared to all contrasts between males and female subgroups. All values represent Cohen's d values (females - males). Parcels are colored by cortical types (left). Source data are provided as a Source Data file.

Only female donor.

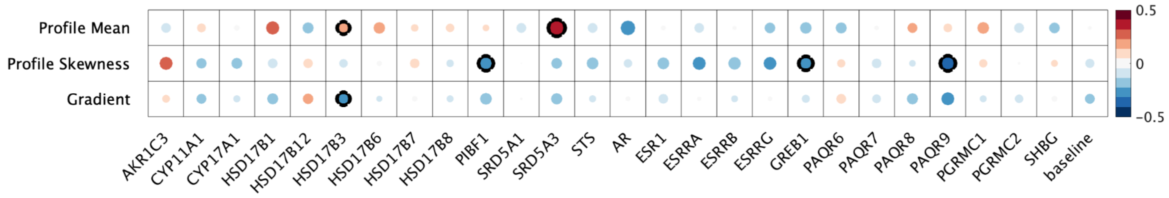

Only male donors.

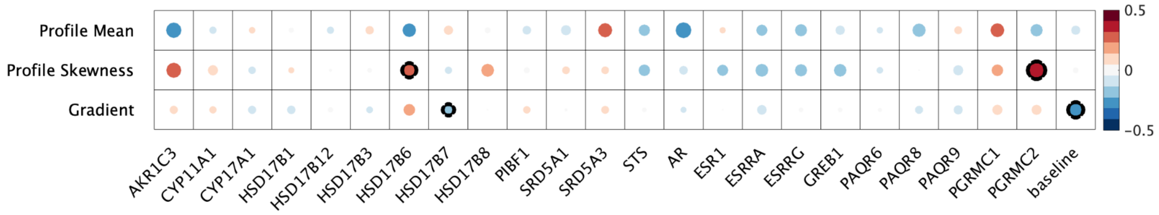

Supplement 10. Spatial overlap between effect maps of sex differences for the microstructural gradient, profile mean and profile skewness, split by AHBA donor-sex. Top and bottom are the same analysis, but considering only the female (top, n=1) and male (bottom, n =5) AHBA donors to derive the transcriptomic maps. Transcriptomic maps of genes are sorted by categories: sex hormone synthesis related genes, androgen receptor related, estrogen receptor related genes, and progesterone receptor related genes. We test for spatial specificity by comparing against the principal component of all genes (baseline). Shades of red represent positive r-values, shades of blue represent negative correlations; circle size and shading indicate size of correlation. p-values < 0.05 after correcting for auto-correlations using spin-testing are marked with a black outline. Source data are provided as a Source Data file.

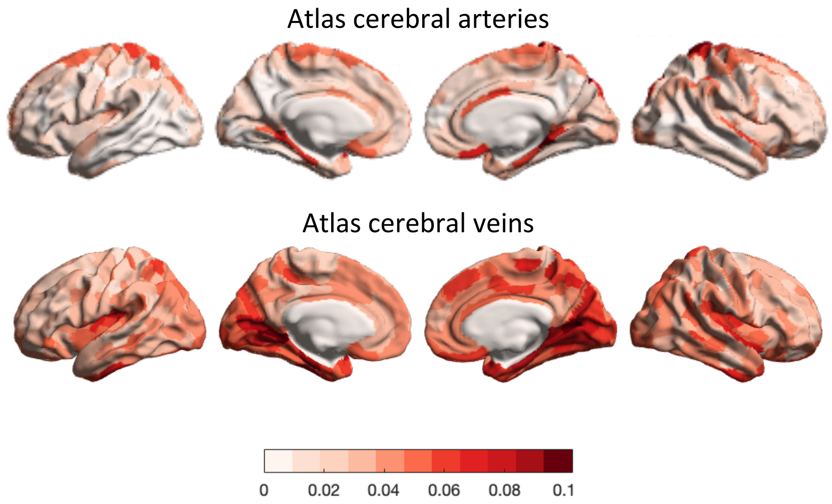

Supplement 11. Atlas of cerebral artery and vein density. Darker shades of red symbolise a higher density of arteries or veins, respectively. Source data are provided as a Source Data file.

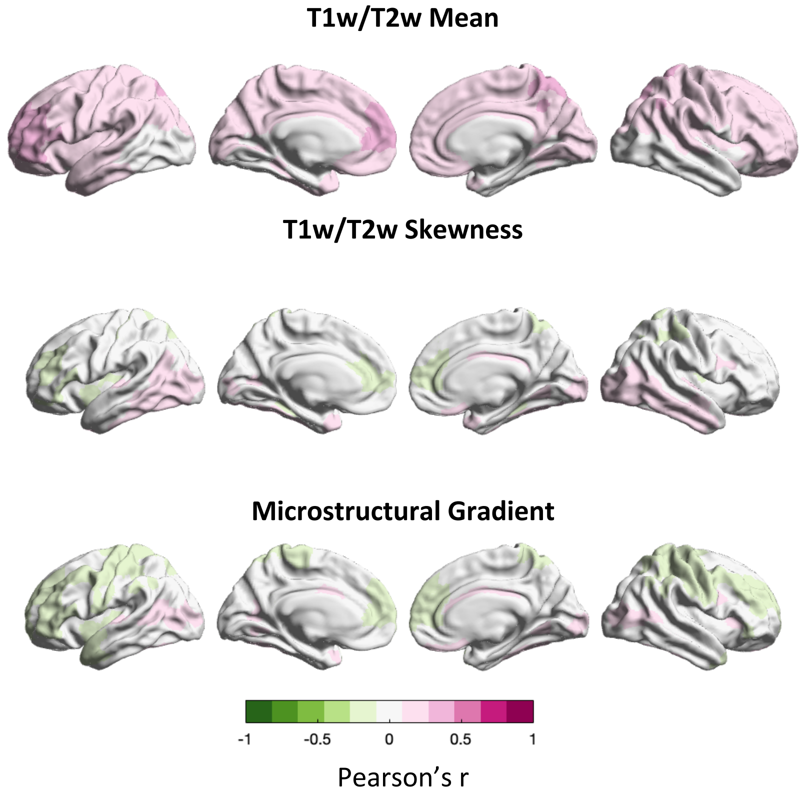

Supplement 12. Intracortical T1w/T2w signal intensity profiling, correlation with ICV. Parcel-wise correlation between ICV per subject (n = 1093) and microstructural measures across the cortex. Pink reflects positive, green reflects negative correlations. No value is higher than r = .34 for T1w/T2w

mean, the peak value for T1w/T2w skewness is r = .21, and the highest correlation between ICV and a gradient parcel is -.21. Source data are provided as a Source Data file.

Genes	Gradient corr	**Gradient** p	**Gradient** pspin	**Mean** corr	**Mean** p	**Mean** pspin	**Skew** corr	**Skew** p	**Skew** pspin
AR	-0.0434	0.5529	0.385	**-0.3119**	**0.0000**	**0.201**	-0.1071	0.1425	0.341
ESR1	-0.0198	0.7864	0.413	0.0442	0.5455	0.36	**-0.1817**	**0.0125**	**0.05**
ESRRA	-0.1278	0.0797	0.213	-0.1522	0.0366	0.329	**-0.2382**	**0.0010**	**0.164**
ESRRB	-0.1540	0.0345	0.068	0.0085	0.9073	0.446	**-0.2141**	**0.0032**	**0.046**
ESRRG	-0.0475	0.5155	0.386	-0.1573	0.0307	0.33	**-0.2166**	**0.0028**	**0.229**
GREB1	-0.0230	0.7534	0.378	-0.0799	0.2743	0.318	**-0.2379**	**0.0010**	**0.016**
PAQR6	-0.0078	0.9150	0.471	-0.0488	0.5047	0.455	-0.0132	0.8568	0.469
PAQR7	-0.0559	0.4442	0.24	0.0047	0.9482	0.508	-0.0665	0.3628	0.226
PAQR8	-0.1597	0.0283	0.097	-0.1683	0.0207	0.27	-0.0261	0.7216	0.431
PAQR9	-0.1462	0.0447	0.116	0.1191	0.1025	0.265	-0.1922	0.0082	0.096
PGRMC1	0.1108	0.1289	0.201	**0.2616**	**0.0003**	**0.171**	0.1771	0.0149	0.184
PGRMC2	-0.0305	0.6771	0.414	0.1360	0.0620	0.161	0.0278	0.7043	0.402
AKR1C3	0.1181	0.1055	0.216	**-0.2922**	**0.0000**	**0.106**	**0.3137**	**0.0000**	**0.054**
CYP11A1	0.0795	0.2767	0.2	-0.0347	0.6355	0.39	0.1135	0.1199	0.162

Genes	Gradient corr	**Gradient** p	**Gradient** pspin	**Mean** corr	**Mean** p	**Mean** pspin	**Skew** corr	**Skew** p	**Skew** pspin
AR	-0.0434	0.5529	0.385	**-0.3119**	**0.0000**	**0.201**	-0.1071	0.1425	0.341
ESR1	-0.0198	0.7864	0.413	0.0442	0.5455	0.36	**-0.1817**	**0.0125**	**0.05**
CYP17A1	-0.0825	0.2590	0.156	0.0420	0.5662	0.343	-0.0940	0.1980	0.121
HSD17B1	-0.1096	0.1333	0.127	0.0858	0.2401	0.234	0.0207	0.7768	0.453
HSD17B12	-0.0192	0.7926	0.419	-0.1027	0.1595	0.329	0.0104	0.8868	0.539
HSD17B3	-0.1184	0.1045	0.063	**0.1276**	**0.0803**	**0.044**	0.0126	0.8632	0.436
HSD17B6	0.1316	0.0711	0.116	-0.1630	0.0251	0.144	0.2089	0.0040	0.11
HSD17B7	**-0.1242**	**0.0885**	**0.044**	0.1192	0.1024	0.053	0.0010	0.9894	0.475
HSD17B8	0.0006	0.9938	0.495	-0.0331	0.6512	0.396	0.1692	0.0200	0.145
PIBF1	-0.0401	0.5832	0.372	0.0000	0.9998	0.53	**-0.2544**	**0.0004**	**0.026**
SRD5A1	-0.1348	0.0645	0.14	0.0920	0.2077	0.316	-0.1469	0.0438	0.17
SRD5A3	0.0130	0.8594	0.404	**0.3135**	**0.0000**	**0.065**	-0.0114	0.8758	0.473
STS	-0.0341	0.6406	0.389	-0.1431	0.0496	0.325	-0.1972	0.0066	0.203

Supplementary table 1. Genetic decoding, all results. Spatial overlap between effect maps of sex differences for the microstructural gradient, profile mean and profile skewness**.** The table shows correlations and their respective p values, and spin-corrected p-values (one-sided; determined by where the empirical r-value falls in the distribution of 1000 random spherical spin-permutations). Transcriptomic maps of genes are of the following categories: sex hormone synthesis related genes, androgen receptor related, estrogen receptor related genes, and progesterone receptor related genes.
