# Supplementary material for: Relating sex-bias in human cortical and hippocampal microstructure to sex hormones
Source: Nat Commun. 2024 Aug 23;15:7279. doi: 10.1038/s41467-024-51459-7 (PMC11344136; doi:10.1038/s41467-024-51459-7)
Supplement: Supplementary file 3 — Reporting Summary [file 41467_2024_51459_MOESM3_ESM.pdf]

nature portfolio

Corresponding author(s): Svenja Küchenhoff

Last updated by author(s): 21/06/2024

Reporting Summary

Nature Portfolio wishes to improve the reproducibility of the work that we publish. This form provides structure for consistency and transparency in reporting. For further information on Nature Portfolio policies, see our Editorial Policies and the Editorial Policy Checklist.

Statistics

For all statistical analyses, confirm that the following items are present in the figure legend, table legend, main text, or Methods section.

n/a	Confirmed	
☐	☒	The exact sample size (*n*) for each experimental group/condition, given as a discrete number and unit of measurement
☐	☒	A statement on whether measurements were taken from distinct samples or whether the same sample was measured repeatedly
☐	☒	The statistical test(s) used AND whether they are one- or two-sided<br>*Only common tests should be described solely by name; describe more complex techniques in the Methods section.*
☐	☒	A description of all covariates tested
☐	☒	A description of any assumptions or corrections, such as tests of normality and adjustment for multiple comparisons
☐	☒	A full description of the statistical parameters including central tendency (e.g. means) or other basic estimates (e.g. regression coefficient) AND variation (e.g. standard deviation) or associated estimates of uncertainty (e.g. confidence intervals)
☐	☒	For null hypothesis testing, the test statistic (e.g. *F*, *t*, *r*) with confidence intervals, effect sizes, degrees of freedom and *P* value noted<br>*Give P values as exact values whenever suitable.*
☒	☐	For Bayesian analysis, information on the choice of priors and Markov chain Monte Carlo settings
☐	☒	For hierarchical and complex designs, identification of the appropriate level for tests and full reporting of outcomes
☐	☒	Estimates of effect sizes (e.g. Cohen's *d*, Pearson's *r*), indicating how they were calculated

Our web collection on statistics for biologists contains articles on many of the points above.

Software and code

Policy information about availability of computer code

Data collection	Data analysed in our results were obtained from the open-access HCP S1200 young adult sample (HCP; http://www.humanconnectome.org/).
Data analysis	All code is costum written in Matlab or Python and is publically available here: https://github.com/svennikue/sex-hormones-x-cortical-structure.git<br>We included the following openly available toolboxes in our analysis:<br>Freesurfer (FSaverage5) for data preprocessing. BrainSpace (Vos de Wael et al., 2020) for gradient computation. HippUnfold (DeKraker et al., 2022) for hippocampal segmentation and unfolding. SurfStat (Worsley, 2009) for linear mixed effect model estimation. Abagen (Zenodo) and Brainstat (2021) for genetic decoding. ENIGMA toolbox for spin-permutation testing (Larivière, S., Paquola, C., Park, By. et al. The ENIGMA Toolbox: multiscale neural contextualization of multisite neuroimaging datasets. Nat Methods 18, 698–700 (2021). https://doi.org/10.1038/s41592-021-01186-4).

For manuscripts utilizing custom algorithms or software that are central to the research but not yet described in published literature, software must be made available to editors and reviewers. We strongly encourage code deposition in a community repository (e.g. GitHub). See the Nature Portfolio guidelines for submitting code & software for further information.

Data

Policy information about availability of data

All manuscripts must include a data availability statement. This statement should provide the following information, where applicable:

- Accession codes, unique identifiers, or web links for publicly available datasets
- A description of any restrictions on data availability
- For clinical datasets or third party data, please ensure that the statement adheres to our policy

This study followed institutional review board guidelines of corresponding institutions. All data analyzed in this study is publicly available. MRI data were obtained from the open-access HCP S1200 young adult sample (HCP: http://www.humanconnectome.org/). We accessed transcriptomic maps provided by the Allen Human Brain Atlas (AHBA) via the BrainStat and abagen toolboxes. Atlases used for the histological analyses were made available by the Dutch Connectome Lab (http://www.dutchconnectomelab.nl/economo/), and Bernier et al. (2018, https://github.com/braincharter/vasculature). We further provide source data within this paper, and make all code available in the project's Github repository (https://github.com/svennikue/sex-hormones-x-cortical-structure.git).

Research involving human participants, their data, or biological material

Policy information about studies with human participants or human data. See also policy information about sex, gender (identity/presentation), and sexual orientation and race, ethnicity and racism.

Reporting on sex and gender	The sample used in the study is the HCP S1200 young adult data release 147. Here, a 'gender' and a 'menstruation' variable have been assessed based on self-report.<br>In this study, we examine the effect of sex hormones and biological sex. We thus classified individuals of female sex if they self-reported their gender as female and indicated that they are or have been menstruating in their lives, and as male if they self-report their gender as male and indicate that they have not been menstruating.
Reporting on race, ethnicity, or other socially relevant groupings	Not relevant to this study, but included in the HCP dataset (see below).
Population characteristics	Here we investigate young (pre- menopause) adults, the age mean +- SD was 28.8 +- 3.7 years (age range = 22-37 years). The dataset used has a strong family structure: n = 298 monozygotic, n = 188 dizygotic twins, n = 449 not related individuals, which we considered as a controle in a supplementary analysis to avoid confounds.<br>We included individuals for whom the scans and data had been released after passing the HCP quality control and assurance standards. The full set of inclusion and exclusion criteria are described elsewhere. Briefly, he HCP dataset includes functional and structural MRI data acquired with 3T scanners from a total of 1206 healthy adult twins and their non-twin siblings born in Missouri, as well as behavioral and cognitive measures and extensive demographic and health-related data. Participants were recruited based on data from the Missouri Department of Health and Senior Services Bureau of Vital Records. The HCP consortium aimed at collecting a representative sample in respect to behavioral, ethnic, and socioeconomic diversity. To allow for sufficient variability in the healthy sample, only severe neurodevelopmental, neuropsychiatric and cardiovascular illnesses were excluded.
Recruitment	Recruitement was done by the HCP. They report that "White non-Hispanic, Hispanic, Asian and African-American families will be invited to participate, to reflect the ethnic diversity of America. Many of the families will include twin pairs, who may be either monozygotic (genetically identical) or dizygotic/fraternal (genetically no more related than ordinary full siblings)". We accounted for family structure in a supplementary analysis and show that they don't affect the results.
Ethics oversight	The Independent Research Ethics Committee at the Medical Faculty of the Heinrich-Heine-University of Duesseldorf (study number 2018-317) approved of using the open dataset and analysis in the context of the current study.

Note that full information on the approval of the study protocol must also be provided in the manuscript.

Field-specific reporting

Please select the one below that is the best fit for your research. If you are not sure, read the appropriate sections before making your selection.

☒ Life sciences ☐ Behavioural & social sciences ☐ Ecological, evolutionary & environmental sciences

For a reference copy of the document with all sections, see nature.com/documents/nr-reporting-summary-flat.pdf

Life sciences study design

All studies must disclose on these points even when the disclosure is negative.

Sample size	The current study was an explorative and multi-level study. Hence, for the first two questions; sex bias and hormonal links in microstructure we used HCPS1200, testing robustness though random subsampling. This is one of the few large-scale datasets that includes micro-structural data (T1wT2w) and (self-reported) hormonal status in (largely) healthy young adults. For the latter two questions we combined this data with independent datasets of transcriptomics (varying subsamples to test robustness) and vessel architecture.<br>Out of the 1206 available HCP datasets, we removed all subjects with missing MRI values, so that we included n = 1093 individuals (n = 298 monozygotic, n = 188 dizygotic twins, n = 449 not related individuals), out of which n = 594 were female. The age mean +- SD was 28.8 +- 3.7 years (age range = 22-37 years).

	Out of the initial 1206 subjects, we overall included n = 867 subjects (n = 500 female, n = 367 male) in the hippocampal analysis. Lastly, for the hormonal anlysis, n = 170 women indicated they took oral contraceptives, and n = 284 were included in the high or low estrogen and progesterone menstrual cycle phase groups, since they indicated that they had regular menstrual cycles.
Data exclusions	We removed individuals with missing structural imaging data for the main analysis. We removed females who indicated that they did not have a regular menstrual cycle or that their menstrual cycle was longer than 29 days for the hormonal analysis. We excluded n = 160 subjects with anatomical anomalies or tissue segmentation errors, n = 93 subjects for which no preprocessed T1w images were available, and n = 86 with morphological outliers (thickness, surface area, gyrification, curvature or T1w/T2w values exceeding 2.5STD of group average) for the hippocampal analysis.
Replication	Split-half reliability analysis based on random permutations within the HCPS1200 release. We demonstrate good reliability for profile mean and skewness, and reasonable replicability for the gradient, as well as very good internal consistency for the hippocampus (Supplement 5). We demonstrate good internal consistency for different male sub-samples for the hormonal contrasts (Supplement 8). We also repeated the sex-difference analysis successfully, controlling for family structure and cortical thickness (Supplement 3). For the transcriptomic analysis, we show reasonable correlations with our results if only the female or male donors are considered (Supplement 10).
Randomization	We controlled for age and intracranial volume in all analyses. We include an additional control analysis in the supplement where we repeated the analysis of all three measures regressing out cortical thickness and including the family structure (interaction between zychosity and family status) as a random effect to demonstrate that our results were not affected by these variables. We also add a control analysis where we report the correlation between sex-difference effects and cerebral vasculature.
Blinding	Blinding was not relevant to this study as grouping was based on participant information.

Reporting for specific materials, systems and methods

We require information from authors about some types of materials, experimental systems and methods used in many studies. Here, indicate whether each material, system or method listed is relevant to your study. If you are not sure if a list item applies to your research, read the appropriate section before selecting a response.

Materials & experimental systems

n/a	Involved in the study	
☒	☐	Antibodies
☒	☐	Eukaryotic cell lines
☒	☐	Palaeontology and archaeology
☒	☐	Animals and other organisms
☒	☐	Clinical data
☒	☐	Dual use research of concern
☒	☐	Plants

Methods

n/a	Involved in the study	
☒	☐	ChIP-seq
☒	☐	Flow cytometry
☐	☒	MRI-based neuroimaging

Plants

Seed stocks	*Report on the source of all seed stocks or other plant material used. If applicable, state the seed stock centre and catalogue number. If plant specimens were collected from the field, describe the collection location, date and sampling procedures.*
Novel plant genotypes	*Describe the methods by which all novel plant genotypes were produced. This includes those generated by transgenic approaches, gene editing, chemical/radiation-based mutagenesis and hybridization. For transgenic lines, describe the transformation method, the number of independent lines analyzed and the generation upon which experiments were performed. For gene-edited lines, describe the editor used, the endogenous sequence targeted for editing, the targeting guide RNA sequence (if applicable) and how the editor was applied.*
Authentication	*Describe any authentication procedures for each seed stock used or novel genotype generated. Describe any experiments used to assess the effect of a mutation and, where applicable, how potential secondary effects (e.g. second site T-DNA insertions, mosiacism, off-target gene editing) were examined.*

Magnetic resonance imaging

Experimental design

Design type	Structural MRI scan only (anatomical), T1w and T2w
Design specifications	n/a
Behavioral performance measures	n/a

Acquisition

Imaging type(s)	T1-weighted (T1w) and T2-weighted (T2w) structural scans (0.7mm isotropic)
Field strength	3 Tesla
Sequence & imaging parameters	Two T1w and two T2w images were collected in a total of 32 minutes, using identical parameters respectively. T1w was acquired with the 3D MPRAGE sequence (Mugler III & Brookeman, 1990) in 256 sagittal slices with an echo time of 2.14ms, an inversion time of 1000ms, and a repetition time of 2400ms (flip angle = 8°; matrix = 320). The T2w images with identical geometry as the T1w ones were acquired with the turbo spin-echo sequence (Mugler et al., 2000) allowing for variable flip angles, with an echo time of 565 ms, a repetition time of 3200 ms, and a bandwidth of 744 Hz per pixel.
Area of acquisition	whole brain field of view

Diffusion MRI ☐ Used ☒ Not used

Preprocessing

Preprocessing software	We worked with readily preprocessed data. Data was preprocessed with the Freesurfer version 5.3. Amongst other steps, T1w and T2w images were co-registered, corrected for field bias, segmented and their ratio (T1w/T2w) was projected to the cortical surface in FSaverage5. Detailed pipelines and preprocessing steps are described in (Glasser et al., 2013)
Normalization	please see above
Normalization template	Individual subject space and averaged Schaefer parcels
Noise and artifact removal	please see above
Volume censoring	please see above

Statistical modeling & inference

Model type and settings	We modelled the principal microstructural gradient using non-linear dimensionality reduction techniques (diffusion embedding, brainspace.readthedocs.io). We computed the skewness and mean microstructural profile measure from the 12 datapoints per parcel which resulted from the 12 equivolumetric surfaces between the pial and white matter surface (FreeSurfer).<br>The linear model for the sex difference analysis + hormonal subgroups was:<br>T1w/T2w measure (parcel) ~ b0 * 1 + b1 * sex + b2 * age + b3 * ICV<br>In the supplement, we furthermore repeated the analysis of all three measures but regressing out cortical thickness and including the family structure (interaction between zychosity and family status) as a random effect.
Effect(s) tested	Sex difference analysis: females - males contrast, two-sided t-statistics, FDR-correction and Cohen's d<br>Hormonal groups: females - males contrast, two-sided t-statistics, FDR-correction and Cohen's d; and to determine whether effect sizes differed per group comparison, we computed a one-way ANOVA on the Cohen's d values across the 400 parcels between groups and post-hoc contrasts based on Tukey's honestly significant difference procedure<br>Genetic decoding: spearman correlations between gene expression maps and sex difference t-statistic maps; spin-permutation test to account for spatial autocorrelations and spearman correlation with principal component (PCA) of all combined gene maps to account for gene specificity.<br>Histological decoding: pin-test based spearman correlations between cortical types and sex difference t-statistic maps<br>Vascular-hormonal coupling: pin-test based spearman correlations between cortical vein and artery density maps.

Specify type of analysis: ☒ Whole brain ☐ ROI-based ☐ Both

Statistic type for inference<br>(See Eklund et al. 2016)	We did not use voxel-wise or cluster-wise statistics, but ran all analyses on the 400 Schaefer parcels and on hippocampal vertices.
Correction	Sex difference + hormonal sub-group analyses: We two-sidedly corrected the t-values for a false discovery rate (FDR) of .05 (Benjamini et al., 2005; Benjamini & Hochberg, 1995). For easier comparison between tests, we report the effect size quantified by Cohen's d for all results that reached significance after this defined FDR-correction threshold.<br>Genetic decoding, histological decoding, vascular-hormonal coupling: spin-permutation-tests where the unthresholded three phenotypic maps (t-statistics of sex differences per measure) were randomly spun in 1000 permutations and correlated with the respectiv spatial maps (genes, histology, vasculature). We report the frequency in which the correlation between permuted phenotypic maps exceeded the original test statistic as spin-p-value. We also provide FDR-threshold where multiple tests occur.

Models & analysis

n/a	Involved in the study
☒	☐ Functional and/or effective connectivity
☒	☐ Graph analysis
☒	☐ Multivariate modeling or predictive analysis
